# Fulminant systemic capillary leak syndrome due to C1 inhibitor deficiency complicating acute dermatomyositis: a case report

**DOI:** 10.1186/1752-1947-8-28

**Published:** 2014-01-27

**Authors:** Ilse Gradwohl-Matis, Romana Illig, Hermann Salmhofer, Daniel Neureiter, Andreas Brunauer, Martin W Dünser

**Affiliations:** 1 Department of Anesthesiology, Perioperative Medicine and General Intensive Care, Paracelsus Private Medical University and Salzburg General Hospital, Müllner Hauptstrasse 48, 5020 Salzburg, Austria; 2 Institute of Pathology, Paracelsus Private Medical University and Salzburg General Hospital, 5020 Salzburg, Austria; 3 Internal Medicine I, Paracelsus Private Medical University and Salzburg General Hospital, 5020 Salzburg, Austria

**Keywords:** Acute heart failure, Angioedema, C1 inhibitor deficiency, Dermatomyositis, Shock, Systemic capillary leak

## Abstract

**Introduction:**

Dermatomyositis is a chronic inflammatory disorder characterized by muscular and dermatologic symptoms with variable internal organ involvement. This is the first report on a patient with acute dermatomyositis and fulminant systemic capillary leak syndrome.

**Case presentation:**

A 69-year-old Caucasian woman with chronic dermatomyositis presented with clinical signs of severe hypovolemic shock and pronounced hemoconcentration (hematocrit, 69%). Her colloid osmotic pressure was 4.6mmHg. Following a bolus dose of prednisolone (500mg), fluid resuscitation was initiated. During volume loading, anasarca and acute respiratory distress rapidly developed. Echocardiography revealed an underfilled, hypokinetic, diastolic dysfunctional left ventricle with pericardial effusion but no signs of tamponade. Despite continued fluid resuscitation and high-dosed catecholamine therapy, the patient died from refractory shock 12 hours after intensive care unit admission. A laboratory analysis of her complement system suggested the presence of C1 inhibitor deficiency as the cause for systemic capillary leakage. The post-mortem examination revealed bilateral pleural, pericardial and peritoneal effusions as well as left ventricular hypertrophy with patchy myocardial fibrosis. Different patterns of endomysial/perimysial lymphocytic infiltrations adjacent to degenerated cardiomyocytes in her myocardium and necrotic muscle fibers in her right psoas major muscle were found in the histological examination.

**Conclusions:**

This case report indicates that acute exacerbation of chronic dermatomyositis can result in a fulminant systemic capillary leak syndrome with intense hemoconcentration, hypovolemic shock and acute heart failure. In the presented patient, the cause for diffuse capillary leakage was most probably acquired angioedema, a condition that has been associated with both lymphoproliferative and autoimmunologic disorders.

## Introduction

Dermatomyositis is a chronic inflammatory disorder characterized by muscular and dermatologic symptoms with variable internal organ involvement [1]. Although the disease progresses with intermittent episodes, most acute dermatomyositis exacerbations do not result in critical illness. Organ dysfunction occurs only in rare cases. While sepsis is a common cause for intensive care unit admission in these patients, respiratory distress and cardiac complications may require organ support [2].

## Case presentation

A 69-year-old Caucasian woman with monoclonal gammopathy of uncertain significance and chronic dermatomyositis (first diagnosed 5 years ago; pretreated with azathioprine 50mg/day and methylprednisolone 6mg daily following acute exacerbations; azathioprine therapy was stopped months before this admission) presented with weakness as well as muscle and joint pain of recent onset. On clinical examination, signs of hypovolemic shock (arterial lactate, 3.7mmol/L; base deficit, –8.9mmol/L; oliguria; extensive skin mottling; tachycardia, 140 beats/minute; tachypnea, 30 breaths/minute; absent venous filling; systolic/mean/diastolic arterial blood pressure, 75/55/40mmHg; low mixed venous oxygen saturation, 35 to 45%; and cardiac index, 0.9–1.2L/minute/m^2^) were present. The laboratory analyses were remarkable for a hematocrit of 69%, serum creatinine of 1.6mg/dL, colloid osmotic pressure of 4.6mmHg, a serum myoglobin concentration of 336μg/L and a serum creatine kinase of 173IU/L. Fluid resuscitation was started and an intravenous bolus dose of prednisolone (500mg) administered. Transthoracic echocardiography revealed an underfilled, diffusely hypokinetic (ejection fraction using the monoplane Simpson’s method, 35%), diastolic dysfunctional left ventricle with pericardial effusion but no signs of tamponade. Based on these findings, a dobutamine (5.5μg/kg/minute) and subsequently an epinephrine (up to 0.86μg/kg/minute) infusion was started. Fluid resuscitation was continued (Ringer’s lactate, 10.500mL; 4% gelatine, 1.000mL; 20% albumin, 100mL) but only led to transient increases of arterial blood pressure and ventricular filling in echocardiography. The hematocrit remained at 62% while lactate levels (9.5mmol/L), base deficit (-16.7mmol/L) and skin mottling worsened. Clinical and echocardiographic signs of intravascular hypovolemia persisted. By that time, the patient had developed anasarca and acute respiratory distress with hypoxemia despite high-flow oxygen inhalation and a short period of non-invasive ventilation. Orotracheal intubation was difficult due to diffuse laryngeal/pharyngeal swelling. Despite continued fluid resuscitation and high-dosed catecholamine therapy, she died from refractory shock 12 hours after intensive care unit admission.

**Figure 1:**
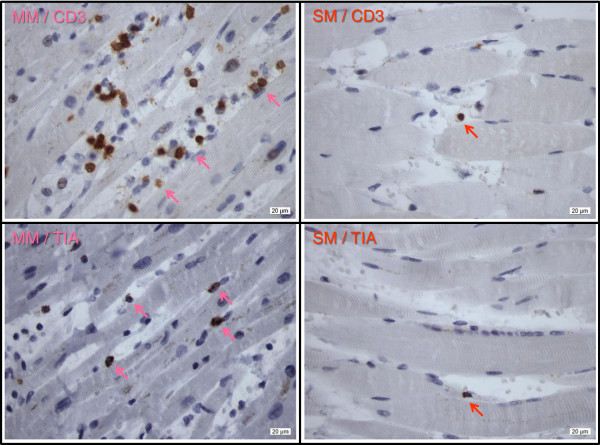
**Immunohistochemical staining of CD3 and T-cell intracellular antigen in myocardial muscle and skeletal muscle.** Arrows indicate positive expression of brownish-stained lymphocyte cells embedded within bluish myocardiocytes and skeletal muscle cells, respectively. Abbreviations: MM, myocardial muscle; SM skeletal muscle; TIA, T-cell intracellular antigen.

Table 1 displays the laboratory results of the complement analyses which were taken shortly after intensive care unit admission but analyzed only post-mortem. The same blood sample was analyzed for frequent auto-antibodies (antinuclear, anti-smooth muscle, anti-mitochondrial, anti-glomerular, perinuclear-antineutrophil cytoplasmic antibody (ANCA), cytoplasmic-ANCA, proteinase 3, and myeloperoxidase); the results were found to be negative. The autopsy revealed bilateral pleural, pericardial and peritoneal effusions as well as left ventricular hypertrophy with patchy myocardial fibrosis. Different patterns of endomysial/perimysial lymphocytic infiltrations (Table 2) adjacent to degenerated cardiomyocytes in her myocardium and necrotic muscle fibers in her right psoas major muscle (Figure 1) were found in the histological examination. No indications for a septic focus or concomitant malignancy were observed.

**Table 1:** Laboratory results of the complement analyses

**Parameter**		**Normal range**
C3 Complement	14mg/dL	86–184mg/dL
C4 Complement	<6mg/dL	10–40mg/dL
C1 Inhibitor	14mg/dL	18–32mg/dL
C1 Inhibitor activity	34%	70–130%

**Table 2:** Summary of perimysial/endomysial expression pattern of applied immunohistochemical markers in assessed myocardial and skeletal muscle tissue samples

**Applied marker**	**Myocardial muscle**	**Skeletal muscle**
CD^3^	**+ + / +**	**+ / –**
CD^4^	**+ + / +**	**+ / –**
CD^8^	**+ / –**	**– / –**
CD^20^	**– / –**	**– / –**
CD^25^	**– / –**	**– / –**
CD^38^	**+ / –**	**– / –**
CD^56^	**+ / –**	**– / –**
CD^79a^	0 / 0	0 / 0
T-cell intracellular antigen-1	**+ / +**	0 **/ –**

Legend to symbols: **++** = high expression, **+** = moderate expression, 0 = sporadic expression, **–** = no expression.

Legend to Table 2: Immunohistochemical expression pattern of lymphocytic specific markers revealed more frequent expression of CD3 in myocardial than in skeletal muscle tissue. In addition, within the CD3 positive T-cells CD4 positive cells were higher expressed than CD8 positive cells indicating a majority of the T-cell-helper-phenotype (CD4^+^) than of the T-cell-regulatory-phenotype (CD8^+^). This was supported by the perimysial expression of CD38 – a marker for activated CD4^+^-T-cells; and the lack of CD25 – a marker for activated CD8^+^-T-cells. Finally, evidence for an activated innate immune system was provided by the specific expression of CD56 and T-cell intracellular antigen (specific marker for a subpopulation of natural killer cells). Overall, immunohistochemical expression analysis revealed a myocardial-associated lymphatic-disordered status in dermatomyositis.

## Discussion

Although limb edema and anasarca have been reported in acute dermatomyositis [3], a systemic capillary leak syndrome, as present in our patient, has not been described before. The initial clinical presentation with hyperlactatemia, oliguria, skin mottling and absent venous filling suggested that hypovolemia was the leading clinical problem. The high hematocrit (69%), representing severe hemoconcentration, together with the acute disease onset without any obvious external loss of water makes excessive fluid loss due to capillary leakage the most probable explanation for hypovolemic shock. A colloid osmotic pressure of 4.6mmHg further suggests loss of macroproteins, such as albumin, occurred over the vascular barrier. This is likely to have reversed the transcapillary oncotic gradient and aggravated transendothelial fluid shift and anasarca. One may speculate that infusion of more colloids, particularly albumin solutions, instead of crystalloids would have expanded intravascular volume to a larger extent and resulted in less tissue edema. However, in the presence of capillary leakage, colloids are equally filtered into the interstitial space and may thus even intensify tissue edema.

Another component of cardiovascular failure in our patient, which only became clinically evident during fluid resuscitation, was acute heart failure. Echocardiographically, heart failure was caused by reduced systolic and diastolic pump function and remained unresponsive to beta-mimetic stimulation. The histologic post-mortem analysis revealed autoimmune myocarditis which can explain the clinical picture of combined systolic and diastolic heart failure. Pericardial effusion was most probably a sign of generalized transcapillary fluid loss and serositis. While cardiac involvement in patients with dermatomyositis (e.g. myocarditis, conduction abnormalities, coronary artery disease) is frequent [4,5], only a few reports on acute heart failure or pericardial tamponade in these patients have so far been published [4-7].

At presentation, sepsis and anaphylaxis were considered possible differential diagnoses for shock. However, the fact that the patient had experienced three similar episodes with hemoconcentration (but no shock symptoms) before, the presence of low inflammatory parameters (C-reactive protein, 2.3mg/dL; leukocytes, 14G/L), absence of a clinically obvious infectious focus and no potential allergen exposition made sepsis and anaphylaxis extremely improbable causes of shock in our patient. Hemophagocytic lymphohistiocytosis has been reported as a cause for multiple organ failure in acute dermatomyositis [8]. Although we did not determine serum ferritin or perform a bone marrow puncture in our patient, the absence of bi-/pancytopenia, elevated liver enzymes or triglyceride levels makes hemophagocytic lymphohistiocytosis improbable. An overlap syndrome with other autoimmune conditions as well as the presence of any malignancy, such as intravascular lymphoma, was excluded both by laboratory results and the post-mortem analysis.

The laboratory evaluation of the complement system (Table 1) strongly suggests the diagnosis of angioedema due to C1 inhibitor deficiency in our patient. Given a negative family history of angioedema and its first presentation at an age >65 years, acquired angioedema was probably present in this case. Acquired angioedema is frequently associated with lymphoproliferative disorders such as non-Hodgkin’s lymphoma but has been reported with autoimmunologic diseases, too [9]. Altered B-cell proliferation control is one proposed link between autoimmunity and lymphoproliferation [10]. Interestingly, some months before this hospital admission, our patient was diagnosed with monoclonal gammopathy of uncertain significance, a well-known potential precursor of B-cell lymphoproliferative disorders [11].

The pathogenesis of angioedema is increased vascular permeability due to excessive bradykinin generation resulting from activation of the complement and contact system due to the lack of C1 inhibitor activity. Triggering events for angioedema vary widely and include any acute disease, physical and psychological stress [9]. One may speculate that exacerbation of dermatomyositis could have been the triggering event in our patient. Nonetheless, the clinical presentation in the presented case was atypical for angioedema which characteristically presents with facial swelling, upper airway edema and abdominal complaints due to intestinal wall edema. So far, angioedema has only once been reported in conjunction with dermatomyositis. Narasimhan *et al.* described the case of a 6-year-old boy with hereditary angioneurotic edema which was associated with juvenile dermatomyositis [12]. Acute treatment of hereditary and acquired angioedema consists of intravenous administration of C1 inhibitor concentrate. Alternatively and in emergency cases, fresh frozen plasma, which contains C1 inhibitor as well, can be transfused [9,13]. In view of the atypical presentation of angioedema in our patient, C1 inhibitor concentrates were not empirically administered although this may have, in retrospect, reversed the capillary leak and that might have saved the patient’s life.

Another interesting aspect of our patient’s disease course was that myoglobin serum concentrations were only mildly elevated whereas creatine kinase values remained within the normal range. This is in contrast to the typical clinical presentation of patients with acute dermatomyositis [1] and has been described in the literature as dermatomyositis sine myositis or amyopathic dermatomyositis [14,15]. Interestingly, this subphenotype seems to be more common than previously thought and may even affect a relevant portion of dermatomyositis cases [14]. Patients with amyopathic dermatomyositis carry the same risk of fatal complications (e.g. interstitial lung disease, malignancy) as patients with myopathic dermatomyositis [14]. Our case confirms these observations.

## Conclusions

This case report indicates that acute exacerbation of chronic dermatomyositis can result in fulminant systemic capillary leak syndrome with intense hemoconcentration, hypovolemic shock and acute heart failure. In the presented patient, the cause for diffuse capillary leakage was most probably acquired angioedema, a condition that has been associated with both lymphoproliferative and autoimmunologic disorders.

## Consent

Written informed consent was obtained from the next-of-kin (husband) of the deceased patient for publication of this case report and accompanying images. A copy of the written consent is available for review by the Editor-in-Chief of this journal.

## Competing interests

The authors declare that they have no competing interests.

## Authors’ contributions

IGM and MWD were involved in the care of the patient and drafted the manuscript. RI and DN performed the pathologic and histologic examinations and revised the manuscript for important intellectual content. HS and AB were involved in the care of the patient and revised the manuscript for important intellectual consent. All authors read and approved the final manuscript.
